# RETRACTION: Astragaloside IV Ameliorates Fat Metabolism in the Liver of Ageing Mice Through Targeting Mitochondrial Activity

**DOI:** 10.1111/jcmm.70876

**Published:** 2025-09-28

## Abstract

**RETRACTION:** LuoZ. , WangY. , XueM. , XiaF. , ZhuL. , LiY. , JiaD. , ChenS. , XuG. and LeiY. , “Astragaloside IV Ameliorates Fat Metabolism in the Liver of Ageing Mice Through Targeting Mitochondrial Activity,” Journal of Cellular and Molecular Medicine 25, no. 18 (2021): 8863–8876, 10.1111/jcmm.16847.34402182 PMC8435431

The above article, published online on 17 August 2021 in Wiley Online Library (wileyonlinelibrary.com), has been retracted by agreement between the journal Editor‐in‐Chief, Stefan N. Constantinescu; the Foundation for Cellular and Molecular Medicine; and John Wiley & Sons Ltd. The retraction has been agreed upon following an investigation into concerns raised by a third party. The investigation revealed duplication between western blot bands presented in Figures 3A and 4B, where the duplicated bands represented different proteins. Furthermore, the IB: anti‐β‐actin bands in Figure 3A were found in an earlier publication by a different group of authors. The authors cooperated with the investigation and explained the duplication was a result of figure mismanagement. They also provided some supporting data; however, it did not correspond to the published figures. Given the nature of the concerns, the editors consider the results and conclusions of this article to be unreliable. The authors disagree with the retraction.

**RETRACTION:** Z. Luo , Y. Wang , M. Xue , F. Xia , L. Zhu , Y. Li , D. Jia , S. Chen , G. Xu and Y. Lei , “Astragaloside IV Ameliorates Fat Metabolism in the Liver of Ageing Mice Through Targeting Mitochondrial Activity,” Journal of Cellular and Molecular Medicine 25, no. 18 (2021): 8863–8876, 10.1111/jcmm.16847.34402182 PMC8435431

The above article, published online on 17 August 2021 in Wiley Online Library (wileyonlinelibrary.com), has been retracted by agreement between the journal Editor‐in‐Chief, Stefan N. Constantinescu; the Foundation for Cellular and Molecular Medicine; and John Wiley & Sons Ltd. The retraction has been agreed upon following an investigation into concerns raised by a third party. The investigation revealed duplication between western blot bands presented in Figures 3A and 4B, where the duplicated bands represented different proteins. Furthermore, the IB: anti‐β‐actin bands in Figure 3A were found in an earlier publication by a different group of authors. The authors cooperated with the investigation and explained the duplication was a result of figure mismanagement. They also provided some supporting data; however, it did not correspond to the published figures. Given the nature of the concerns, the editors consider the results and conclusions of this article to be unreliable. The authors disagree with the retraction.

